# Substitute Behaviors following Residential Substance Use Treatment in the Western Cape, South Africa

**DOI:** 10.3390/ijerph182312815

**Published:** 2021-12-05

**Authors:** Deborah Louise Sinclair, Steve Sussman, Maarten De Schryver, Cedric Samyn, Sabirah Adams, Maria Florence, Shazly Savahl, Wouter Vanderplasschen

**Affiliations:** 1 Department of Psychology, University of the Western Cape, Cape Town 7535, South Africa; mflorence@uwc.ac.za; 2 Department of Special Needs Education, Ghent University, 9000 Ghent, Belgium; Cedric.Samyn@UGent.be (C.S.); wouter.vanderplasschen@ugent.be (W.V.); 3 Department of Population and Public Health Sciences, University of Southern California, Los Angeles, CA 90032, USA; ssussma@usc.edu; 4 Faculty of Psychology and Educational Sciences, Faculty Research Support Office, Ghent University, 9000 Ghent, Belgium; maarten.deschryver@ugent.be; 5 Centre for Higher Education Development, Language Development Group, University of Cape Town, Cape Town 7701, South Africa; sabirah.adams@uct.ac.za; 6 Centre for Interdisciplinary Studies of Children, Families and Society, University of the Western Cape, Cape Town 7535, South Africa; ssavahl@uwc.ac.za

**Keywords:** substitute behaviors, recovery, substance use, behavioral addictions, substance use treatment

## Abstract

The dynamics of substitute behaviors and associated factors remain poorly understood globally, and particularly in low- and middle-income contexts. This prospective study describes the prevalence and types of substitute behaviors as well as predictors, correlates, and motivations associated with substitution in persons (n = 137) admitted to residential substance use treatment in the Western Cape province of South Africa. The brief assessment of recovery capital, overall life satisfaction scale, and an adapted version of the addiction matrix self-report measure were completed during and post-treatment. Results indicate that substitutes were employed consciously for anticipated appetitive effects, for time-spending, (re)connecting with others, and enjoyment. At follow-up, 36% of service users had substituted their primary substance(s) with another substance or behavior; 23% had relapsed and 40% had maintained abstinence. While some service users may be especially vulnerable to developing substitute behaviors, targeted prevention and intervention efforts can reduce this risk.

## 1. Introduction

Substitute behaviors are an important aspect to be taken into account by persons in addiction recovery and the organizations and services supporting these individuals [1]. While a universally agreed-upon definition remains elusive [2], there is a longstanding recognition that other behaviors or addictions may arise while abstaining from a primary substance [2,3,4,5,6], particularly during early recovery (1–12 months) [7,8,9]. A recent scoping review in which substitute addictions were defined as the “immediate or gradual functional replacement of an addiction or set of addictions that have been terminated”, underscores that substitute behaviors may become addictions and display key characteristics of addictive behaviors ([10], p. 692). Consequently, substitute behaviors are part of a continuum where behaviors have the potential to progress to addictive levels over time and which vary in severity. When these behaviors are a purposeful component of treatment (e.g., nicotine replacement therapy; methadone maintenance treatment) these should not be regarded as substitute addictions [10].

Following abstinence from a primary substance, compensatory behaviors may emerge due to forced abstinence [11], curiosity and experimentation [12], and when potential substitutes are available and accessible and expected to provide certain effects [13]. Prior experience with (potential) substitutes may foster these expectations, as may perceptions of its safety and adverse effects [1,7,12,14]. (Un)consciously ‘selected’ substitutes may be initiated or resumed during the life course [15,16,17]. In treatment samples, substitution may co-occur with and continue when abstaining from a primary substance, fulfilling similar function(s) [7,9,18]. Research findings among inpatient and residential samples point to a subset of persons with a substance use disorder (SUD) who initiate or resume the use of other substances [15,16,17], substitute with behaviors including gambling, compulsive eating, and work [19,20] and/or relapse to their primary substance [21]. Despite the variety of treatment goals [22] and the fact that short-term substitute behaviors may be promotive of recovery [1], each substitute for a previous/latent addiction increases the risk of relapse [7,23]. Although relapse remains possible throughout the recovery process [24], its likelihood is particularly high immediately post-treatment [25] and in early recovery [26]. Yet, few studies have focused on the prevalence, correlates, and motives for substitute behaviors in the emerging addiction recovery literature.

The extant literature on substitute behaviors in persons with SUDs demonstrates varying conceptions of its onset (e.g., during or after treatment), nature (e.g., substance or behavior), function (e.g., relapse prevention), and duration (e.g., short- or long-term) [10]. The primary focus lays on substance-to-substance substitution [10], but substance-to-behavior substitutions, encompassing DSM-5-listed disorders as well as behaviors subjectively experienced as addictions without diagnostic criteria (e.g., compulsive sex, shopping, and exercise [27]), have rarely been examined. Behavioral substitutes for alcohol that have been reported include compulsive work, hobbies, gambling; mystical belief, prayer, and meditation; increased involvement with religion and Alcoholics Anonymous [8,19]. Based on a recent scoping review, the prevalence of substitution in substance use treatment samples is estimated between 7% and 92% (despite differences in conceptualization, operationalization, and sample size [10]). Correlates of substitute behaviors include greater severity and duration of substance use, comorbid mental health problems, younger age, and male gender [28,29].

In low- and middle-income countries (LMICs), a few case studies have shown that pornography viewing, and increased technology use can substitute for substance addictions [18,30,31,32]. LMICs such as South Africa are characterized by disproportionately high rates of SUDs driven by social, behavioral, policy and legislative factors, but these countries face significant structural and logistic barriers and huge treatment gaps [33,34,35]. Alongside the limited focus on behavioral substitutes in persons with SUDs, the literature on substitution has paid very little attention to recovery capital, which is increasingly recognized as a crucial element for initiating and maintaining recovery [36,37]. Recovery capital, encompassing personal, family/social, and community resources that support recovery [36], and quality of life (QoL) are important indicators of stable recovery [37]. Understanding its associations with substitute behaviors/addictions will inform the further development of recovery-oriented support services.

The overarching aim of this study was to determine the prevalence, correlates and motives of substitute behaviors after initial treatment among individuals with SUDs in the Western Cape, South Africa. Specific objectives included:

- To establish the prevalence and types of substitutes.
- To determine the correlates of substitute behaviors.
- To explore the underlying motives for substitute behaviors.

Although a few recent studies have addressed this topic [12,38,39], none included the wide array of potential (substance and behavioral) substitutes considered in this study. To our knowledge, this is also the first study to empirically examine substitute behaviors in a LMIC context, i.e., South Africa. Considering the high rates of SUDs and related harm, low treatment entry, and high post-treatment relapse in this country [40,41,42], knowledge on the prevalence of substitute addictions and associated personal and contextual factors is essential to optimize the organization of treatment and recovery-oriented support [9,10,25].

## 2. Materials and Methods

### 2.1. Overview

The study employed a longitudinal cohort design, with study assessments at baseline and after 3 months. The baseline study sample (n = 207) was purposively drawn from a cohort of consecutively admitted persons with SUDs receiving residential treatment in five facilities in South Africa. The criteria for including these treatment facilities were full or partial funding from the National Department of Social Development; location (the Western Cape province) and delivery of a specialized, residential program for SUDs. Though these facilities were alike in their philosophy based on total abstinence and the services offered, they varied concerning program duration and capacity. Three facilities were single-gender services. Questionnaires were administered to respondents at two time points: during and following treatment. The follow-up period ranged from 63 to 294 days, with a mean of 168 days and a median of 163 days (SD = 44.647). Baseline data were collected between 21 June 2019 and 16 September 2019, while follow-up data were collected between 15 October 2019 and 31 March 2020. Table 1 summarizes the main characteristics of the treatment facilities.

To be eligible, service users were required to (1) be 18 years of age or older; (2) be in treatment for a minimum of 2 weeks; (3) have signed a consent form; (4) not exhibit acute psychotic symptoms, and (5) agree to be interviewed at follow-up. In total, 207 respondents agreed to participate during the recruitment period. To receive specialist treatment in these facilities, service users are required to present with a SUD. Written informed consent was obtained at baseline for conducting the baseline and follow-up interviews. The study was approved by the Biomedical Research Ethics Committee of the University of the Western Cape (BM18/4/13) and the Western Cape Department of Social Development (Reference: 12/1/2/4).

### 2.2. Measures

The baseline and follow-up assessments contained questions on socio-demographic background, the Brief Assessment of Recovery Capital [43], an adapted version of the Addiction Matrix Self-report Measure [44], and the Overall Life Satisfaction scale [45]. Follow-up interviews contained the same measures but did not reassess demographic variables. The baseline assessment additionally comprised questions regarding substance use history (primary substance and other substances used) and contact information to enable successful follow-up. Additional questions posed at follow-up included whether the baseline treatment episode was completed and whether (and which, if any) substances had been used post-treatment. All questionnaires were translated into Afrikaans and back-translated into English. An Afrikaans mother-tongue speaker, a service provider (senior social worker) at one of the participating facilities, tested and approved the final translated version. The data were collected through interviewer-administered baseline and follow-up questionnaires, which facilitated the collection of more detailed and complex data [46]. All baseline assessments were conducted face-to-face at the facilities, while follow-up interviews were administered face-to-face (during home visits and meetings in public spaces; 60%; and telephonically; 40%). All baseline and follow-up interviews were conducted by the first author. A follow-up rate of 66.2% (n = 137) was achieved. Reasons for loss to follow-up were unwillingness to participate (n = 22); obsolete or incomplete contact information (n = 20); failed efforts to reach respondents (n = 17); being missing/whereabouts unknown (n = 4); displaying acute psychiatric symptoms (n = 3); being institutionalized (n = 3) or death (n = 1).

Demographic variables included in the study were: respondents’ self-reported age (in years); gender (male/female/other); race (coded as ‘Black African’, ‘Coloured’, ‘Indi-an/Asian’ or ‘White’ (in apartheid South Africa, the racial categories ‘Black African’, ‘Coloured’ and ‘Indian/Asian’ were assigned to those denied the same benefits as ‘Whites’ to reinforce segregation. Their use here is only for descriptive purposes, given the importance of ongoing redress efforts)); relationship status (single; in a committed relationship; married; cohabiting; divorced/separated/widowed) and whether respondents lived with a partner that used substances; their highest level of education (primary school/secondary school/post-secondary) and employment status (employed/unemployed/prospect of employment post-treatment).

#### 2.2.1. The Brief Assessment of Recovery Capital (BARC-10)

Abridged from the Assessment of Recovery Capital scale [47], the BARC-10 [43] is a 10-item measure of recovery, individual and social assets. Each item of the BARC-10 is scored on a scale from 1 (strongly disagree) to 6 (strongly agree), with higher scores indicative of more recovery capital. The BARC-10 has been found to be psychometrically sound, with good concurrent validity with the original 50-item ARC (r = 0.92; [43]). Predictive validity has been demonstrated for ≥1-year abstinence with a cut-off score of 47 (the sumscore) [43]. Given the profile of the study respondents, a unipolar rather than a bipolar response format was chosen (1 = not at all agree; 2 = agree a little; 3 = agree somewhat; 4 = agree a lot and 5 = agree completely), as unipolar response formats are considered less cognitively demanding [48]. As such, scores could vary from 10 to 50, with higher scores indicating higher levels of recovery capital. Internal consistency for the current sample was α = 0.75.

#### 2.2.2. The Addiction Matrix Self-Report Measure

This 30-item measure taps various addictive behaviors through one item for each type of potential addiction [49]. Participants are asked to endorse three statements (use, addiction, and period) at baseline concerning several potentially addictive behaviors, referring to the 14 days preceding treatment: “I used/did it before treatment” is scored on a 5-point Likert scale (0 = never/1 = seldom/2 = sometimes/3 = often/4 = very often); the statement “I was ‘addicted’ to it before treatment” is also scaled on a 5-point Likert scale (ranging from 0 = not at all agree/1= agree a little/2 = agree somewhat/3 = agree a lot/4 = agree completely) and to specify in years and months “For how long?”. Twenty-nine potential addictions were listed as response categories and a 30th item enabled an open-ended response to indicate any other substance or behavioral addiction. To understand the motives underlying potential substitution, respondents were asked in two open-ended questions why they had increased (if any) some behaviors: “Why do you think you’ve increased the use of other substances since leaving treatment?” and “Why do you think there’s been an increase in certain types of your behaviors since leaving treatment?”

The original Addiction Matrix Self-report Measure was adapted to include substances used in the Western Cape as reflected in treatment demand data [50]. This adaptation process centrally involved persons in recovery. Revisions included refining the descriptions of what behaviors encompassed, removing items as well as integrating substances known to be used among treatment-seekers in the region. For example, the item originally worded *other drugs* (such as cocaine, stimulants, hallucinogens, XTC, opiates, Valium or others) was separated and detailed; rather than *stimulants*, *crystal methamphetamine* and *methcathinone* (*CAT*) were specified; *LSD* replaced *hallucinogens* and *inhalants* were removed. *Heroin* and *nyaope*/*whoonga* were exchanged for *opiates* and *methaqualone* (*Mandrax*) was added. *Eating* (way too much food each day, binge eating) was modified to include ‘high-sugar foods such as chocolates’ and ‘purging’ as well as ‘food restriction’ were also included. The item *gambling* (including slot machines, casino games, lotteries, scratch cards, online) was also modified to include betting on horse racing and sports, a legal mode of gambling known to frequently occur in the study context [51]. The original item *sex* was revised to encompass sexual activity, pornography use, voyeurism as well as online sexual activity. Candidate items were then subjected to cognitive interviewing [52]. As access to service users was not permitted for these cognitive interviews, this process was undertaken with three addiction counselors in recovery employed at one of the residential treatment facilities. Feedback from this process informed the phrasing and refinement of the questionnaire.

#### 2.2.3. The Overall Life Satisfaction Scale (OLS)

Increased well-being and QoL are important elements of addiction recovery, alongside abstinence, and therefore, also core treatment objectives [53]. The OLS, as a validated 1—item measure of QoL, was administered at baseline and follow-up. The OLS measure, composed of the statement “Overall, how satisfied are you with your life as a whole?”, is scored on a scale from 0 (not at all satisfied) to 10 (completely satisfied) and has been found to be a reliable indicator of QoL [45]. 

### 2.3. Statistical Analyses

All statistical analyses were performed using R version 4.0.4 [54]; alpha was set at *p* < 0.05 prior to all analyses. Descriptive statistics were generated for socio-demographic data and substance-related characteristics of the baseline and follow-up sample. 

A binary logistic regression model was used to examine the impact of study attrition (n = 70; 34%). Presence in the follow-up study (coded 0 = not in the follow-up, and 1 = in the follow-up) was defined as the outcome variable, while the variables gender, relationship status, living with a partner using substances and primary substance, together with the covariate age, were defined as predictors. These predictors were theoretically and empirically motivated [55]. The analysis revealed no significant effects between those retained and those not retained in the study. As such, there is no evidence that both samples differ concerning the predictors gender, relationship status, living with a partner that uses substances, primary substance(s), and the covariate age. Full information maximum likelihood procedures, argued to yield equivalent results to multiple imputation, were used for missing values in the remaining analyses [56]. Three post-treatment outcome categories were constructed. Relapse was operationalized as any post-treatment use of the primary substance(s), while abstinence referred to no use of the primary substance(s). Substitution was operationalized as an increase in the use of a substance and/or engagement in behavior(s) in addition to self-perceived addiction following abstinence from the primary substance(s). The decision that ‘agree a lot’ or ‘agree completely’ were indicative of addiction emanated from dialogues with two persons in stable recovery (41 and 26 years, respectively, and one of whom worked for participating services) and discussion within the research team. There is no universally accepted definition or terminology for substitute behaviors [10], but central features are that substitution may be conscious or unconscious; may involve substances and/or behaviors; that abstinence (rather than a reduction) of the primary substance(s) is necessary; that there should be an increase in the new behavior and functional replacement of the terminated addiction and that a substitute behavior may be initiated (newly acquired) or resumed. Two independent coders assessed all cases individually, based on these criteria. The level of intercoder agreement, calculated using Cohen’s kappa, yielded almost perfect agreement (k = 0.926; [57]).

Given the limited literature on substitute behaviors, a binary logistic regression model was constructed to predict ‘substitution’ (objective one). Being in the Substitute Group (coded 0 = not in the substitute group, and 1 = in the substitute group) was defined as the outcome variable, while the variables gender, relationship status, employment status, living with a partner that used substances (pre-treatment) and primary substance and the covariates age, recovery capital (BARC-10 post-treatment) and QoL (OLS post-treatment) were defined as predictors. Significant effects are described using fitted values and 95% confidence intervals as described in the R packages “effects” [58]. As the first model did not distinguish between persons who abstained and relapsed (i.e., those not in the substitute group), this can be considered as a heterogeneous group—and higher within-group variance might be expected.

To examine objective two, a multinomial log-linear model via neural networks [59] was fit to the data with outcome category (abstinence/relapse/substitution) as the outcome variable and the factors gender, relationship status, employment status, living with a partner that used substances and primary substance and the covariates age, recovery capital, and QoL as predictors. To facilitate interpretation of the effects, a more parsimonious model was fit to the data with outcome category as the outcome variable and the three significant predictors (living with a partner that used substances, primary substance, and recovery capital (BARC-10 post-treatment). Next, a new dataset was created with all possible combinations of the two factors (living with a partner that used substances and primary substance) and the covariate recovery capital (BARC post-treatment). There were three values chosen for the scores on the BARC-10: a low score (mean value (39.58) minus twice the standard deviation (6.61), the mean score, and a high score (the mean value plus twice the standard deviation). This resulted in a dataset containing 30 data points (2 × 5 × 3) for which the model predicted membership probability. That is living with a partner that uses substances represents 2 levels (yes/no), primary substance(s) accounts for 5 levels (alcohol, crystal methamphetamine, heroin, Mandrax or other) and the 3 BARC-10 values result in 2 × 5 × 3 = 30 possible combinations (e.g., partner “no”, alcohol, BARC-10 score of 6.61).

To explore objective three, open-ended, qualitative responses in the questionnaire were analyzed thematically. Results are presented as set out in the objectives of the study.

## 3. Results

### 3.1. Study Sample

The study sample (n = 137) comprised 87 (63.5%) men and 50 (36.5%) women (see Table 2). Respondents ranged in age from 18 to 67 years (SD = 9.88), with a mean age of 32.1 years. Most respondents were single (51.1%) and not living with a partner that used substances (66.4%). Before entering treatment, most respondents were unemployed (47.5%). Crystal methamphetamine (56.9%) was reported to be the most widely used primary substance at treatment admission, followed by alcohol, other substances (CAT, cannabis, cigarettes, and cocaine), heroin, and Mandrax. That respondents often identified multiple primary substances is likely indicative of polysubstance use. However, the extent to which these substances and other behaviors were engaged simultaneously, and in which sequencing and quantity are unknown.

### 3.2. Post-Treatment Outcomes: Relapse, Abstinence, and Substitute Behaviors

In line with objective one of the study, 50 cases were found to have substituted (36.5%), 55 (40.1%) to have abstained, and 32 (23.4%) to have relapsed at follow-up. The proportion of the group sizes of the outcome categories ‘Abstained’, ‘Relapsed’ and ‘Substituted’ differed significantly (χ^2^(2) = 6.41, *p* < 0.041). ‘Relapsed’ was defined as any period of resuming use of the primary substance(s) after discharge, regardless of duration, or proportion of the total time post-discharge. ‘Abstained’ corresponded to no reported use of the primary substance(s) following discharge from treatment. ‘Substituted’ indicated that use of the primary substance(s) had not been resumed post-treatment; that other substances were used and/or behaviors were engaged, and that there was a subjectively perceived addiction to the replacement/s as indicated by the response ‘agree a lot’ or ‘agree completely’ to the question of whether they were addicted to the behavior/substance in the last 30 days.

### 3.3. Substitute Behaviors

Among those reporting substitute behaviors (n = 50), 21 respondents reported multiple substitutes. Leading replacements for the primary substance were love (e.g., thoughts, feelings, behaviors about love and relationships) (n = 24); caffeine (e.g., coffee, or energy drinks such as Red Bull) (n = 11); eating (way too much food each day and/or high-sugar foods such as chocolates; binging; purging; food restriction) (n = 9); exercise (e.g., sports/extreme sports) (n = 8); cigarettes (n = 8); social media (e.g., Facebook, Twitter, Instagram, WhatsApp) (n = 7) and religion (activities/practices) (n = 7). Six persons reported work and binge-watching (e.g., TV series, movies, documentaries) as a substitute. Sex (e.g., sexual activity, pornography use, voyeurism, online), self-harm (cutting, skin picking, hair pulling), compulsive internet use (surfing the web), and online or offline gaming (e.g., PlayStation, Xbox, Wii) were only reported by two respondents. Finally, compulsive shopping (in stores; online), alcohol, and cannabis addiction were found in only three single cases.

In terms of objective two, the results of the binary logistic regression analysis revealed a significant effect of employment status (χ^2^(2) = 6.03, *p* = 0.049) and living with a (licit or illicit) substance using partner (χ^2^(2) = 4.28, *p* = 0.039) on substitute behaviors. Based on the estimated effects, it was found that participants in the category ‘prospect of employment’ had a higher probability of being in the Substitute Group ($\hat{\pi }$ = 0.53, 95 % CI = [0.35, 0.70]) when compared to those employed ($\hat{\pi }$ = 0.25, 95 % CI = [0.13, 0.45]) or unemployed ($\hat{\pi }$ = 0.29, 95 CI = [0.18, 0.42]). Participants not living with a partner that used substances ($\hat{\pi }$ = 0.41, 95 % CI = [0.31, 0.53]) were more likely to be in the Substitute Group than those who did ($\hat{\pi }$ = 0.21, 95 % CI = [0.11, 0.38]).

The multinomial log-linear model found significant predictors for living with a partner using substances (χ^2^(2) = 6.29, *p* = 0.042), primary substance (χ^2^(8) = 17.55, *p* = 0.025) and recovery capital (χ^2^(2) = 8.96, *p* = 0.011). For instance, the average predicted membership probabilities for someone living with a partner using substances are 0.36 to have abstained, 0.39 for relapse, and 0.26 for substitution. In contrast, for someone living with a non-using partner, these probabilities are 0.34, 0.22 and 0.45, respectively. These numbers suggest that one is more likely to substitute when not living with a partner using substances. Persons using heroin and Mandrax as the primary substance had a higher probability of substituting when compared to persons who used alcohol and crystal methamphetamine as primary substances. Lower scores on recovery capital were linked to a higher likelihood of relapse, while high scores were associated with a higher probability of abstinence. The likelihood of substituting was highest for those with medium scores on the BARC-10. Aggregated probabilities are summarized in Table 3.

### 3.4. Motives for Substitution

In line with objective three, respondents’ responses to the two open-ended questions of why their use of other substances and/or certain types of behaviors increased since leaving treatment revealed several underlying factors.

The most salient motives involved using substitutes consciously to replace a primary substance and for its anticipated effects (e.g., for energy; to cope; to manage cravings; n = 22), for time-spending (e.g., to occupy time; due to boredom; n = 16), to (re)connect with others (e.g., improved relationships; to keep contact; to receive recovery support; n = 9), for enjoyment (e.g., interested in; for enjoyment or upliftment; n = 8), as well as unconsciously (e.g., did not know why; unconscious process; n = 8). Other reasons for substitution were availability and access which also encompassed ease of accessibility, availability of money and/or cost (n = 7), and sobriety (e.g., due to a ‘change from the old way of life’; n = 7). Finally, treatment-related motives included continuing/implementing a behavior from treatment (n = 4), while job-related reasons comprised having a new/more stable job or for livelihood (n = 4); being influenced by others included being influenced/triggered by others (n = 2) and for health improvement involved doing an activity for health improvement or because of now being capable of performing a behavior that could not be performed in active addiction (n = 2).

## 4. Discussion

Our findings indicate that substitute behaviors are not uncommon post-treatment among persons who received residential SUD treatment in South Africa and that the majority of substitutes are behaviors rather than substances. Thirty-six percent of the respondents substituted for their SUD in one way or another, while 40% was abstinent and 23% relapsed after treatment. This prevalence rate, though not directly comparable to earlier studies on substitution due to varying operational definitions, sample sizes, treatment experiences, settings and timeframes [10], adds to the growing evidence base on the phenomenon of substitute behaviors following treatment-assisted recovery. Substitute behaviors were diverse, comprising love, caffeine, eating, exercise, cigarettes, and social networking, amongst others, with many participants reporting multiple substitutes. That substitutes were predominantly behavioral (substance-to-behavior-substitution) rather than substances (substance-to-substance-substitution), a category to which those who relapsed also belong, is a key finding for establishing recovery-oriented support and adds to the limited body of knowledge on behavioral substitutes for substance use.

Love emerged as the leading substitute behavior for SUDs in this sample. Sussman ([60], p. 41), who has defined love addiction as “a constricted pattern of repetitive behavior directed toward a love object that leads to negative role, social, safety, or legal consequences”, recognizes that love may substitute for substance use. Love seemingly invokes brain neurotransmission processes similar to substance use and decreases adaptive functioning over time. SUDs are viewed by some as a “committed pathological love relationship … with a mood-altering chemical in expectation of a rewarding experience” ([60], p. 34). This attachment to substance uses concomitantly hinders and replaces interpersonal relationships. While the likelihood of relapse is markedly increased by the substance use of spouses or significant others, interpersonal connections which aid recovery, constituting social recovery capital, are central to addiction recovery [36,61,62]. Our results converge with that of a recent U.S. study [63] investigating the prevalence, co-occurrence, and correlates of substance and behavioral addictions. Love also emerged as the most prevalent addictive behavior among this younger, adolescent sample [63]. Concerns about developing a replacement addiction to love and/or sex [64] underpin the ‘One Year Rule’ of avoiding dating and casual sexual relationships during early recovery and in some treatment settings, for example, therapeutic communities [65,66]. However, an alternative explanation may be that love is representative of service users’ social support from families and broader social networks. Application of the CHIME-D (Connectedness, Hope, Identity, Meaning in life, Empowerment, Difficulties) personal recovery framework has foregrounded the importance of connectedness throughout recovery for 12-step recovery support group members [67,68]. It has also been demonstrated that relapse risk is considerably lower when a spouse or sponsor is supportive of one’s recovery process [61]. Better outcomes have also been reported for service users in relationships that are intact one-year post-treatment. However, partner-related interpersonal stressors and (perceived) substance use problems of the partner have been found to hinder recovery [69].

A second important substitute and replacement substance was caffeine. Caffeinated beverages include coffee, tea, mixed drinks, and energy drinks [70]. The potential for caffeine to be a substitute behavior has long been known [1]. In a 1986 substitution study in the US [71], 56 ‘alcoholics’ in treatment were found to consume significantly more coffee in the first month of treatment than during the six months pre-treatment. Ágoston and colleagues [70] identified six motivational factors for the consumption of caffeinated beverages, namely alertness (eliminating fatigue, enhancing concentration and revitalizing), habit (ritual/ routine), mood (optimizing), social (caffeinated drinks’ importance in social settings), taste (linked to its flavor) and symptom management (e.g., reducing headaches and blood pressure). As caffeine produces dose-dependent symptoms, intoxication may develop with overconsumption, and withdrawal symptoms may emerge with discontinuation [72]. Its psychostimulant properties lead some to become psychologically and physiologically dependent on caffeine [73], as reported by 11 respondents in our study and as suggested by the inclusion of caffeine in the DSM-5.

Food, another prominent substitute in the present study, has been found to differ in its function depending on the stage of recovery. In the U.S., Cowan and Devine’s [74] interviews with 25 males in drug and alcohol recovery found that during the first six months food (particularly sweets and ‘junk’ food) was used as a substitute to control moods, lessen boredom, satisfy cravings and structure days. In Months 7–13 of recovery, the few that used food as a substitute did so to alleviate boredom and/or to satisfy food cravings. During the later stages of recovery (Months 14–36), food was no longer a substitute.

Exercise has been recognized previously as a potential substitute behavior [32]. From an addictive behavior standpoint, exercise is complex to conceptualize and should be distinguished from healthy exercise, which can share attributes with addiction. Exercise addiction may be present as a primary (the main problem) or secondary (as a consequence of a primary problem) symptom [75]. Freimuth, Moniz and Kim [75] distinguish between recreational exercise, at-risk exercise, problematic exercise, and exercise addiction according to the motivation for exercising, consequences, and frequency/control. At the point of exercise addiction, the frequency and intensity of exercise continue, the pleasure diminishes, and the behavior is motivated by avoiding withdrawal symptoms to the impairment of daily functioning and the ability to meet role obligations. Service providers at treatment facilities for SUDs have been cautioned to be aware of the potential of exercising to become addictive, as it may be recommended for its mood-altering effects. Exercising engaged for relieving withdrawal symptoms, as has been reported for cocaine, may open the way for an exercise addiction [75].

Cigarette smoking has been linked to relapse [76] and smoking cessation often has a positive effect on long-term substance use outcomes. Therefore, smoking cessation advice should be offered to those in treatment for SUDs [77]. However, service users and staff frequently smoke cigarettes at treatment services, and treatment programs often do not address tobacco use [78], or consider smoking as a secondary concern [79]. In Friend and Pagano’s [16] study of changes in smoking status during and following substance use treatment, 15% of their sample of 387 persons with alcohol use disorders had initiated smoking during the 12-month follow-up period, often beginning during and increasing significantly after treatment. There have, however, been calls for smoking to be denormalized in SUD treatment settings [80]. Tobacco products may also be used as a coping strategy for withdrawal symptoms experienced during or after SUD treatment [1] or one tobacco product may be used to substitute for another. For example, in a recent study [81], a subgroup of former daily smokers was found to use e-cigarettes for smoking cessation. Other motives included managing nicotine addiction, and avoiding health risks and smoking-related stigma. All participants preferred e-cigarettes over nicotine replacement therapy.

The finding that those with lower recovery capital have a higher probability of relapse is an important component of conceptualizing relapse vulnerability. According to White ([82], p. 30), “most clients entering addiction treatment have never had much recovery capital or have dramatically depleted such capital by the time they seek help”. The positive association between recovery capital scores and substitute behaviors may relate to the availability of human recovery capital and the capacity to apply (alternative, adaptive) coping skills and solve problems in the context of high-risk situations [36]. Treatment intends to build recovery capital by addressing needs that could be detrimental to recovery early on [83].

In terms of socio-demographic factors, those with the prospect of employment had a higher probability of substituting as compared to the employed or unemployed group. One interpretation of this finding could be that having the prospect rather than a guarantee of employment leads to insecurity and stress, prompting substitute behaviors for anticipated effects. Employment is an important need to address and the substance use—employment relationship is complex and reciprocal [84,85,86]. Substance use may negatively impact the return to work or maintenance of a job, while employment may positively or negatively impact substance use behavior [85,86]. As Becker and colleagues ([87], p. 335) note: “unemployment is extremely stressful, but employment can be stressful too”. Unemployment is a significant risk factor for substance use and increases the risk of relapse post-treatment [85]. South Africa’s high rates of unemployment limit prospects of becoming employed [88], particularly post-treatment. On the other hand, employment may be associated with stressors, cues and cravings, new peers who may apply pressure to use substances and greater disposable incomes. These factors also relate to the present study’s finding that the availability of money and/or the cost associated with a behavior as well as the influence of others are motives for substitution. Alongside these potential issues for relapse and substitution, however, it is crucial to acknowledge that employment potentially enhances self-efficacy and social integration, and consequently lowers relapse risk [89].

### Implications and Limitations of the Study

As a goal of substance use treatment is to build (multi-faceted) recovery capital, it is incumbent upon service providers to identify specific needs that may benefit from intervention and to tailor treatment protocols and assessments to service users’ needs. Given that risk or vulnerability is not static, it is imperative for service providers to modify treatment plans and to distinguish the strategies used during early and later treatment stages [7,62,90]. An indispensable component of resolving substitute behaviors is for service users to be sensitized to the possibility that they may arise and that they are equipped to identify if and when further support may be warranted [7,90,91]. The salience of substance-to-behavior-substitution highlights that those treating SUDs must be aware of former or future behavioral addictions. Service providers should also be prepared to address behavioral addictions at treatment entry, especially among persons who have relapsed and/or re-entered treatment. As we have discussed elsewhere [10], substitute behaviors do not necessarily foreshadow a relapse. Substitute behaviors may be an intermediate step towards recovery (see [92]), particularly during early recovery [9,13]. Yet, the nature of the substitute behavior and motives are important to consider in terms of its risk for leading to similar or greater harm, relapse and/or the development of another addiction [9,92,93]. As Freimuth and colleagues ([94], p. 151) caution “any substance or behavior that is reinforcing, used to cope, or provides robust and desired changes in experience has the potential to become an addiction.” This functional replacement role of substitutes has long been recognized [1].

While the current study overcomes shortcomings of earlier empirical work on substitute behaviors, results should be considered in light of some limitations. First, the study was conducted in one geographical area in South Africa and the sample size was relatively small. Though longitudinal studies are critical for studying substitute behaviors, attrition is an established methodological concern. Relapse and substitution may itself be associated with loss to follow up [28]. Second, the end of the follow-up data collection period coincided with a stringent lockdown to contain the COVID-19 pandemic, including a blanket ban on the sale and purchase of cigarettes and alcohol, which is likely to have contributed to altered patterns of use and acquisition, and for some to seek alternatives for the original addiction and substitution [18]. Other possible confounding variables include access to alcohol, though this was reported as a substitute by only a minority of respondents. Third, as data were self-reported, it could be subject to recall and social desirability biases. However, a key strength of the study is the rapport established between the primary researcher and respondents. While appointments often had to be rescheduled on multiple occasions, every effort was made to interview participants where they felt most at ease and had privacy, and in the case of telephonic interviews, when they were most likely to be able to take a call privately so as to feel unconstrained. Respondents were thus able to disclose and detail the dynamics of post-treatment experiences with substances and behaviors more freely, while augmenting the methodological rigor of the study. Finally, as our operationalization of substitution required that there was an increase in use or engagement as well as perceived addiction, it is probable that the range and prevalence of substitutes detected may have differed with another operationalization.

To extend the scientific knowledge base on substitute behaviors as it pertains to treatment-assisted recovery, longer-term follow-up studies should be conducted to establish the trajectory of substitute behaviors. While it is clear that research has been conducted on substitute behaviors over the past decades, there is an urgent need for a framework to unify, systematize, and improve its (variable) quality and to better inform research translation, particularly in LMICs. We also recommend conducting qualitative research into the perceptions and experiences of addiction professionals. Integrating the views of service users and service providers is essential for relevant and responsive treatment.

## 5. Conclusions

Substitute behaviors are a known outcome for some following substance use treatment and targeted interventions may impact its onset, course and outcomes. Service providers should be aware of the risk factors for substitute behaviors, which could aid in identifying service users at high risk and modifying treatment accordingly, such as taking a comprehensive (addiction) history, educating service users and their support networks, and being aware that substitute behaviors may emerge within treatment settings.

## Figures and Tables

**Table 1:** Core features of participating treatment facilities.

Facility	Target Group	Treatment Offered	Duration	Capacity
1	Adult males and females ≥18 years of age	Prevention, individual and group therapy Pharmacological therapy Aftercare	4 weeks (extension possible)	16
2	Adult males ≥18 years of age	Individual and group therapy Pharmacological therapy	12 weeks	30
3	Adult males and females ≥18 years of age	Individual and group therapy Pharmacological therapy	5 weeks	50
4	Adult females ≥18 years of age	Individual and group therapy Pharmacological therapy	9 weeks	30
5	Adult males ≥18 years of age	Individual and group therapy Pharmacological therapy	9 weeks	20

**Table 2:** Characteristics of the follow-up sample (n = 137).

Characteristics	Frequency (n = 137)	%
**Gender**		
Male	87	63.5
Female	50	36.5
**Relationship status**		
Single	70	51.1
In a committed relationship	35	25.6
Married	21	15.3
Cohabiting	11	8.0
**Live with a partner using substances**		
No	12	5.8
Yes	178	86
**Employment status**		
Unemployed	65	47.5
Prospect of employment	37	27.0
Employed	35	25.6
**Primary substance**		
Crystal methamphetamine	78	56.9
Alcohol	19	13.9
Other	18	13.1
Heroin	11	8.0
Mandrax	11	8.0

**Table 3:** Predicted membership probability for abstinence, relapse and substitution.

Predictor	Abstinence (n = 55)	Relapse (n = 32)	Substitution (n = 50)
**Live with a partner using substances**			
No	0.34	0.22	0.45
Yes	0.36	0.39	0.26
**Primary substance**			
Alcohol	0.45	0.44	0.11
Crystal methamphetamine	0.44	0.30	0.26
Heroin	0.20	0.32	0.49
Mandrax	0.13	0.33	0.54
Other	0.53	0.11	0.36
**BARC-10 (follow-up)**			
26.4	0.09	0.58	0.33
39.6	0.33	0.26	0.41
52.8	0.63	0.06	0.32

## Data Availability

Restrictions apply to the availability of these data. Data were obtained from residential treatment facilities and anonymized data are available from the authors with the permission of the Department of Social Development (Western Cape Government, South Africa).

## References

[1] Sussman S., Black D.S. (2008). Substitute Addiction: A Concern for Researchers and Practitioners. J. Drug Educ..

[2] Green J., Jaffe J.H., Carlisi J.A., Zaks A. (1978). Alcohol Use in the Opiate Use Cycle of the Heroin Addict. Int. J. Addict..

[3] Moore M., Raymond A.F., Gray M.G. (1941). Alcoholism and the Use of Drugs; A Review of 841 Cases Diagnosed “with Psychosis Due to Drugs and Other Exogenous Toxins” or “without Psychosis: Drug Addiction. Q. J. Stud. Alcohol.

[4] O’ Donnell J.A. (1964). A Follow-up of Narcotic Addicts; Mortality, Relapse and Abstinence. Am. J. Orthopsychiatry.

[5] Rounsaville B.J., Weissman M.M., Kleber H.D. (1982). The Significance of Alcoholism in Treated Opiate Addicts. J. Nerv. Ment. Dis..

[6] Selby S. (1993). A Look at Cross-Addiction.

[7] Chiauzzi E.J. (1991). Preventing Relapse in the Addictions: A Biopsychosocial Approach.

[8] Murphy S.A., Hoffman A.L. (1993). An Empirical Description of Phases of Maintenance Following Treatment for Alcohol Dependence. J. Subst. Abus. Treat..

[9] White W., Kurtz E. (2006). The Varieties of Recovery Experience: A Primer for Addiction Treatment Professionals and Recovery Advocates. Int. J. Self-Help Self-Care.

[10] Sinclair D.L., Sussman S., Savahl S., Florence M., Adams S., Vanderplasschen W. (2021). Substitute Addictions in Persons with Substance Use Disorders: A Scoping Review. Subst. Use Misuse.

[11] McGlothlin W., Jamison K., Rosenblatt S. (1970). Marijuana and the Use of Other Drugs. Nature.

[12] Shapira B., Berkovitz R., Rosca P., Lev-Ran S., Kaptsan A., Neumark Y. (2021). Why switch? Motivations for self-substitution of illegal drugs. Subst. Use Misuse.

[13] Sussman S., Sussman A.N. (2011). Considering the Definition of Addiction. Int. J Env. Res. Public Health.

[14] Sussman S. (2017). Substance and Behavioral Addictions: Concepts, Causes, and Cures.

[15] De Leon G. (1987). Alcohol Use among Drug Abusers: Treatment Outcomes in a Therapeutic Community. Alcohol. Clin. Exp. Res..

[16] Friend K.B., Pagano M.E. (2004). Smoking Initiation among Nonsmokers during and Following Treatment for Alcohol Use Disorders. J. Subst. Abuse Treat..

[17] Kohn C.S., Tsoh J.Y., Weisner C.M. (2003). Changes in Smoking Status among Substance Abusers: Baseline Characteristics and Abstinence from Alcohol and Drugs at 12-Month Follow-Up. Drug Alcohol Depend..

[18] Sinclair D.L., Vanderplasschen W., Savahl S., Florence M., Best D., Sussman S. (2020). Substitute Addictions in the Context of the COVID-19 Pandemic. J. Behav. Addict..

[19] Vaillant G.E., Milofsky E.S. (1982). Natural History of Male Alcoholism. IV. Paths to Recovery. Arch. Gen. Psychiatry.

[20] Vaillant G.E., Clark W., Cyrus C., Milofsky E.S., Kopp J., Wulsin V.W., Mogielnicki N.P. (1983). Prospective Study of Alcoholism Treatment: Eight-Year Follow-Up. Am. J. Med..

[21] Aharonovich E., Liu X., Samet S., Nunes E., Waxman R., Hasin D. (2005). Postdischarge Cannabis Use and Its Relationship to Cocaine, Alcohol, and Heroin Use: A Prospective Study. Am. J. Psychiatry.

[22] Neale J., Nettleton S., Pickering L. (2011). What Is the Role of Harm Reduction When Drug Users Say They Want Abstinence?. Int. J. Drug Policy.

[23] Melemis S.M. (2015). Relapse Prevention and the Five Rules of Recovery. Yale J. Biol. Med..

[24] Laudet A.B., White W.L. (2008). Recovery Capital as Prospective Predictor of Sustained Recovery, Life Satisfaction, and Stress among Former Poly-Substance Users. Subst. Use Misuse.

[25] Vanderplasschen W., Colpaert K., Broekaert E. (2010). Determinants of Relapse and Re-Admission among Alcohol Abusers after Intensive Residential Treatment. Arch. Public Health.

[26] Gossop M., Stewart D., Marsden J. (2008). Attendance at Narcotics Anonymous and Alcoholics Anonymous Meetings, Frequency of Attendance and Substance Use Outcomes after Residential Treatment for Drug Dependence: A 5-Year Follow-up Study. Addiction.

[27] Sussman S. (2020). The Cambridge Handbook of Substance and Behavioral Addictions.

[28] Kim H.S., Hodgins D.C., Garcia X., Ritchie E.V., Musani I., McGrath D.S., von Ranson K.M. (2021). A Systematic Review of Addiction Substitution in Recovery: Clinical Lore or Empirically-Based?. Clin. Psychol. Rev..

[29] Waldorf D. (1970). Life without Heroin: Some Social Adjustments during Long-Term Periods of Voluntary Abstention. Soc. Probl..

[30] Yousif Ali A. (2015). Substitute Addictions: Catching the Animagi and Throwing Away the Metamorphmagi. Int. J. Emerg. Ment. Health Hum. Resil..

[31] Çepik A., Arikan Z., Boratav C., Lşik E. (1995). Bulimia in a Male Alcoholic: A Symptom Substitution in Alcoholism. Int. J. Eat. Disord..

[32] Tadpatrikar A., Sharma M.K. (2018). Pornography as a Replacement for Substance Use: An Emerging Approach to Understand Addiction Mechanism. Open J. Psychiatry Allied Sci..

[33] Tsuei S.H.-T., Clair V., Mutiso V., Musau A., Tele A., Frank E., Ndetei D. (2017). Factors Influencing Lay and Professional Health Workers’ Self-Efficacy in Identification and Intervention for Alcohol, Tobacco, and Other Substance Use Disorders in Kenya. Int. J. Ment. Health Addict..

[34] Heijdra Suasnabar J.M., Hipple Walters B. (2020). Community-Based Psychosocial Substance Use Disorder Interventions in Low-and-Middle-Income Countries: A Narrative Literature Review. Int. J. Ment. Health Syst..

[35] Semrau M., Alem A., Ayuso-Mateos J.L., Chisholm D., Gureje O., Hanlon C., Jordans M., Kigozi F., Lund C., Petersen I. (2019). Strengthening Mental Health Systems in Low- and Middle-Income Countries: Recommendations from the Emerald Programme. BJPsych Open.

[36] White W., Cloud W. (2008). Recovery Capital: A Primer for Addictions Professionals. Counselor.

[37] Best D., Vanderplasschen W., Nisic M. (2020). Measuring Capital in Active Addiction and Recovery: The Development of the Strengths and Barriers Recovery Scale (SABRS). Subst. Abuse Treat. Prev. Policy.

[38] Koball A.M., Glodosky N.C., Ramirez L.D., Kallies K.J., Gearhardt A.N. (2019). From Substances to Food: An Examination of Addiction Shift in Individuals Undergoing Residential Treatment for Substance Use. Addict. Res. Theory.

[39] Razjouyan K., Hamzenejhad P., Khademi M., Arabgol F. (2018). Data on the Prevalence of Addiction to the Internet among Individuals with a History of Drug Abuse. Data Brief.

[40] Degenhardt L., Glantz M., Evans-Lacko S., Sadikova E., Sampson N., Thornicroft G., Aguilar-Gaxiola S., Al-Hamzawi A., Alonso J., Helena Andrade L. (2017). Estimating Treatment Coverage for People with Substance Use Disorders: An Analysis of Data from the World Mental Health Surveys. World Psychiatry.

[41] Peltzer K., Phaswana-Mafuya N. (2018). Drug Use among Youth and Adults in a Population-Based Survey in South Africa. Afr. J. Psychiatry.

[42] Swanepoel I., Geyer S., Crafford G. (2016). Risk Factors for Relapse among Young African Adults Following In-Patient Treatment for Drug Abuse in the Gauteng Province. Soc. Work. Stellenbosch Online.

[43] Vilsaint C.L., Kelly J.F., Bergman B.G., Groshkova T., Best D., White W. (2017). Development and Validation of a Brief Assessment of Recovery Capital (BARC-10) for Alcohol and Drug Use Disorder. Drug Alcohol Depend..

[44] Sussman S., Arpawong T.E., Sun P., Tsai J., Rohrbach L.A., Spruijt-Metz D. (2014). Prevalence and Co-Occurrence of Addictive Behaviors among Former Alternative High School Youth. J. Behav. Addict..

[45] Cummins R.A., Lau A.D.L. (2005). Personal Wellbeing Index: School Children (PWI-SC).

[46] Meadows K.A. (2003). So You Want to Do Research? 5: Questionnaire Design. Br. J. Community Nurs..

[47] Groshkova T., Best D., White W. (2013). The Assessment of Recovery Capital: Properties and Psychometrics of a Measure of Addiction Recovery Strengths. Drug Alcohol Rev..

[48] Lietz P. (2010). Research into Questionnaire Design: A Summary of the Literature. Int. J. Res. Mark..

[49] Sussman S., Pokhrel P., Sun P., Rohrbach L.A., Spruijt-Metz D. (2015). Prevalence and Co-Occurrence of Addictive Behaviors among Former Alternative High School Youth: A Longitudinal Follow-up Study. J. Behav. Addict..

[50] Dada S., Harker Burnhams N., Erasmus J., Lucas W., Parry C., Bhana A., Pretorius S., Weimann R. (2020). Monitoring Alcohol, Tobacco and Other Drug Abuse Treatment Admissions in South Africa (July - December 2019), SACENDU (South African Community Epidemiology Network on Drug Use).

[51] The National Gambling Board South Africa. https://www.ngb.org.za/organisational-areas/research/statistics/gambling-sector-performance-.aspx.

[52] Drennan J. (2003). Cognitive interviewing: Verbal data in the design and pretesting of questionnaires. J. Adv. Nurs..

[53] Laudet A.B. (2011). The Case for Considering Quality of Life in Addiction Research and Clinical Practice. Addict. Sci. Clin. Pract..

[54] R Core Team (2021). R: A Language and Environment for Statistical Computing (Version 4.0.4.).

[55] Cohen S. (1981). The Effects of Combined Alcohol/Drug Abuse on Human Behavior. Drug Alcohol Abus. Implic. Treat..

[56] Lee T., Shi D. (2021). A Comparison of Full Information Maximum Likelihood and Multiple Imputation in Structural Equation Modeling with Missing Data. Psychol. Methods.

[57] McHugh M.L. (2012). Interrater Reliability: The Kappa Statistic. Biochem. Med. (Zagreb).

[58] Fox J. (2003). Effect Displays in R for Generalised Linear Models. J. Stat. Softw..

[59] Venables W.N., Ripley B.D. (2002). Modern Applied Statistics with S.

[60] Sussman S. (2010). Love Addiction: Definition, Etiology, Treatment. Sex. Addict. Compulsivity.

[61] Ellis B., Bernichon T., Yu P., Roberts T., Herrell J.M. (2004). Effect of social support on substance abuse relapse in a residential treatment setting for women. Eval. Program Plan..

[62] Flores P.J. (2001). Addiction as an Attachment Disorder: Implications for Group Therapy. Int. J. Group Psychother..

[63] Sussman S., Unger J.B., Begay C., Moerner L., Soto C.P. (2021). Co-Occurrence, and Correlates of Substance and Behavioral Addictions among American Indian Adolescents in California. J. Drug Educ..

[64] Zmuda N.A. (2021). No New Relationships? A Study of the Role of Relationships in Relapse among Adults in Early Recovery.

[65] Matesa J. (2016). Sex in Recovery: A Meeting between the Covers.

[66] Vanderplasschen W., Vandevelde S., Broekaert E. (2014). Therapeutic Communities for Treating Addictions in Europe. Evidence, Current Practices and Future Challenges; European Monitoring Centre for Drugs and Drug Addiction. https://www.emcdda.europa.eu/system/files/publications/779/TDXD14015ENN_final_467020.pdf.

[67] Dekkers A., Vos S., Vanderplasschen W. (2020). “Personal Recovery Depends on NA Unity”: An Exploratory Study on Recovery-Supportive Elements in Narcotics Anonymous Flanders. Subst. Abus. Treat. Prev. Policy.

[68] Aga N., Rowaert S., Vander Laenen F., Vandevelde S., Vander Beken T., Audenaert K., Vanderplasschen W. (2021). Connectedness in Recovery Narratives of Persons Labeled Not Criminally Responsible: A Qualitative Study. Int. J. Forensic Ment. Health.

[69] Tracy S.W., Kelly J.F., Moos R.H. (2005). The Influence of Partner Status, Relationship Quality and Relationship Stability on Outcomes Following Intensive Substance-Use Disorder Treatment. J. Stud. Alcohol.

[70] Ágoston C., Urbán R., Király O., Griffiths M.D., Rogers P.J., Demetrovics Z. (2018). Why Do You Drink Caffeine? The Development of the Motives for Caffeine Consumption Questionnaire (MCCQ) and Its Relationship with Gender, Age and the Types of Caffeinated Beverages. Int. J. Ment. Health Addict..

[71] Verinis J.S. (1986). Caffeine Use in the Recovering Alcoholic. Alcohol Health Res. World.

[72] Pohler H. (2010). Caffeine Intoxication and Addiction. J. Nurse Pract..

[73] Addicott M.A. (2014). Caffeine Use Disorder: A Review of the Evidence and Future Implications. Curr. Addict. Rep..

[74] Cowan J., Devine C. (2008). Food, Eating, and Weight Concerns of Men in Recovery from Substance Addiction. Appetite.

[75] Freimuth M., Moniz S., Kim S.R. (2011). Clarifying Exercise Addiction: Differential Diagnosis, Co-Occurring Disorders, and Phases of Addiction. Int. J. Environ. Res. Public Health.

[76] Weinberger A.H., Platt J., Esan H., Galea S., Erlich D., Goodwin R.D. (2017). Cigarette Smoking Is Associated with Increased Risk of Substance Use Disorder Relapse: A Nationally Representative, Prospective Longitudinal Investigation. J. Clin. Psychiatry.

[77] McKelvey K., Thrul J., Ramo D. (2017). Impact of Quitting Smoking and Smoking Cessation Treatment on Substance Use Outcomes: An Updated and Narrative Review. Addict. Behav..

[78] Baca C.T., Yahne C.E. (2009). Smoking Cessation during Substance Abuse Treatment: What You Need to Know. J. Subst. Abus. Treat..

[79] Shu C., Cook B.L. (2015). Examining the Association between Substance Use Disorder Treatment and Smoking Cessation: Substance Use Treatment and Smoking. Addiction.

[80] Schroeder S.A., Morris C.D. (2010). Confronting a Neglected Epidemic: Tobacco Cessation for Persons with Mental Illnesses and Substance Abuse Problems. Annu. Rev. Public Health.

[81] Tokle R., Pedersen W. (2019). “Cloud Chasers” and “Substitutes”: E-cigarettes, Vaping Subcultures and Vaper Identities. Sociol. Health Illn..

[82] White W. (2002). An Addiction Recovery Glossary: The Languages of American Communities of Recovery. Let’s Go Make Some History: Chronicles of the New Addiction Recovery Advocacy Movement.

[83] Cleveland H.H., Brick T.R., Knapp K.S., Croff J.M., Croff J.M., Beaman J. (2021). Recovery and Recovery Capital: Aligning Measurement with Theory and Practice. Family Resilience and Recovery from Opioids and Other Addictions.

[84] Laudet A.B., Humphreys K. (2013). Promoting Recovery in an Evolving Policy Context: What Do We Know and What Do We Need to Know about Recovery Support Services?. J. Subst. Abus. Treat..

[85] Henkel D. (2011). Unemployment and Substance Use: A Review of the Literature (1990-2010). Curr. Drug Abus. Rev..

[86] Richardson L., Epp S. (2016). Substance Use Disorders, employment and the return to work. Handbooks in Health, Work, and Disability.

[87] Becker D.R., Drake R.E., Naughton W.J. (2005). Supported Employment for People with Co-Occurring Disorders. Psychiatr. Rehabil. J..

[88] Naidoo P. South Africa Unemployment Rate Rises to Highest in the World. https://www.bloomberg.com/news/articles/2021-08-24/south-african-unemployment-rate-rises-to-highest-in-the-world.

[89] Graham M.D. (2006). Addiction, the Addict, and Career: Considerations for the Employment Counselor. J. Employ. Couns..

[90] Buga S., Banerjee C., Zachariah F., Mooney S., Patel P., Freeman B. (2017). Cross Addiction-a Case Presentation. Oncol. Hematol. Ro.

[91] Carnes P.J., Murray R.E., Charpentier L. (2005). Bargains with Chaos: Sex Addicts and Addiction Interaction Disorder. Sex. Addict. Compulsivity.

[92] Horvath T.A. (2006). Smart Recovery News & Views.

[93] Moore S.C. (2010). Substitution and complementarity in the face of alcohol-specific policy interventions. Alcohol Alcohol..

[94] Freimuth M., Waddell M., Stannard J., Kelley S., Kipper A., Richardson A., Szuromi I. (2008). Expanding the Scope of Dual Diagnosis and Co-Addictions: Behavioral Addictions. J. Groups Addict. Recovery.

